# Updated chronologies for North American small mammal fossil localities in the Neotoma Paleoecology Database

**DOI:** 10.1038/s41597-025-06491-7

**Published:** 2026-01-27

**Authors:** Val J. P. Syverson, Simon J. Goring, Nicola Cullen, Marta A. Jarzyna, André M. Bellvé, Andrew Martindale, Jessica L. Blois

**Affiliations:** 1 Life and Environmental Sciences, University of California, Merced, Merced, CA USA; 2 Department of Earth and Planetary Sciences, University of California, Davis, Davis, CA USA; 3 Department of GeographyCenter for Climatic ResearchData Science Institute, University of Wisconsin-Madison, Madison, USA; 4 Translational Data Analytics Institute, The Ohio State University, Columbus, Ohio USA; 5 Department of Evolution, Ecology and Organismal Biology, The Ohio State University, Columbus, Ohio USA; 6 School of Environment, Waipapa Taumata Rau, University of Auckland, Auckland, New Zealand; 7 Department of Anthropology, University of British Columbia, Vancouver, BC Canada

**Keywords:** Community ecology, Palaeoecology, Climate-change ecology

## Abstract

Community paleoecology is a powerful approach for analyzing ecological communities during long-term climate shifts like the Pleistocene-Holocene transition, but it depends on accurate estimates of species co-occurrences. The Neotoma Paleoecology Database is an open paleodata resource that stores assemblage-level taxonomic, spatial, and temporal information for Quaternary fossil localities. However, its age estimates for many vertebrate fossil localities are based on uncalibrated radiocarbon dates, hindering comparisons with other paleoenvironmental proxies. In order to provide consistent and updated age inferences suitable for broad-scale paleoecological studies, we have reassessed the radiocarbon chronologies for all 14C-dated North American small mammal collections in Neotoma. Here we present the resulting database update, including 2074 radiocarbon dates newly added to Neotoma and new calibrated radiocarbon chronologies for 1553 fossil collections. The new chronologies cover more sites and include more dates than the chronologies previously available in Neotoma. They also provide fossil assemblage age estimates in calendar years, facilitating integration with other data sources. We anticipate that these updates will be useful for various applications in community paleoecology.

## Background

In order to address complex global ecological questions such as the drivers of past ecosystem change, paleoecological studies increasingly rely on approaches that synthesize data from multiple sites, and that combine multiple data sources and proxy types, such as stable isotopes, pollen counts, macrofossil occurrences, and climate models (e.g.^1–4^). Each proxy, site, and sedimentary archive has its own unique taphonomic pathway. These local- and proxy-scale differences in the fossil record mean that understanding change across spatial scales, a central focus of paleoecology, requires accurate estimates of the ages of fossil samples^5–10^. Aligning fossil data in time across multiple sites and proxies is challenging. A variety of radiometric dating techniques, biostratigraphic markers, tephras, or annual laminations (such as tree rings or varves) can be used to directly estimate sample age, each with their own challenges and biases. Typically, only a subset of samples are dated, then once sample ages are determined, researchers develop an appropriate chronology to infer ages of undated samples based on a range of age modeling methods^6,11–13^. New methods and improvements in both sample age estimation and age modeling continually improve approaches, and different methods will provide slightly different estimates of time^6,12–15^. Additionally, for chronologies based on radiocarbon dates^14,16^, the calibration curves used to translate radiometric age in radiocarbon years before present (^14^C BP) to age in calendar years before present (cal BP) are regularly updated as new information is generated that increases the accuracy and precision of the curves^11,17^. For example, five curves have been developed by the IntCal effort between 1998 and 2020^16,18–22^. Thus, chronologies need to be continually updated and refined to facilitate accurate research inferences.

The challenge of updating chronologies is particularly acute in paleo-data resources that store proxy information from many different sites and studies. One such resource is the Neotoma Paleoecology Database (hereafter “Neotoma”), which is a composite database comprised of 41 constituent databases of biological proxy information, including vertebrate fossils, fossil pollen, ostracodes, diatoms, stable isotopes, and many other proxies^23,24^. Neotoma addresses the challenge of managing changing date calibrations and age interpretation by linking biological proxy data to one or more chronological inferences, which can be updated to incorporate new data, and which may be linked to the individual chronological data used to create them (e.g. radiometric ages, biostratigraphic or archaeological dates). Within Neotoma, *sites* may contain one or more *collection units* (collections), defined as a unit from a site from which a collection of fossils or other data have been made; a single site may contain multiple collection units. A collection unit is typically associated with at least two *datasets*: a dataset of the proxy data associated with samples from different *analysis units* (usually stratigraphic units) within the collection and a dataset of *geochronological ages* associated with samples from some or all of the analysis units within the collection. Direct geochronological ages are linked to the set of observations at a site through a set of *chronological controls* which form the foundation for an overall inferred chronology for the collection unit, including a *sample age* estimate for each analysis unit within the collection unit. The overall collection unit chronology, which merges geochronological information and any other types of chronological information such as biostratigraphic information, tephras, etc. with an age model, provides age estimates for the collection unit overall as well as for each analysis unit within the site^24^.

Each collection unit in Neotoma may be associated with multiple chronologies. One chronology is typically the original chronology developed by individual researchers as part of their work at a site, though additional chronologies may be associated with a collection unit as chronologies are revisited and revised as part of other research efforts^5,25^. Some chronologies may include uncalibrated radiocarbon ages, reported in ^14^C BP, and others may use the calibration curve that was most current at the time of publication. Many collection units have chronologies based only on non-geochronologic dates such as archaeological periods, tephra layers, or biostratigraphic indicators, which may increase uncertainty. Some collection units have no inferred chronology at all, or have chronologies with no reported uncertainty. Overall, there is considerable variation among sites in chronology estimation and quality, which limits potential for broad-scale paleoecological syntheses across space and time.

This challenge is particularly evident in the FAUNMAP database, one of the Neotoma constituent databases, which stores information on Pliocene through Holocene vertebrate fauna of North America^26^. FAUNMAP was a collaborative research effort in the 1990s that produced a standardized set of chronologies associated with vertebrate faunal localities, but where those chronologies were based on radiocarbon dates (Late Pleistocene and later), they were typically provided in ^14^C BP, or a combination of ^14^C BP and cal BP^26,27^. These inconsistencies limit the utility of Neotoma’s chronological information and, where the differences in technique are not clearly labeled, they may mislead researchers. Additionally, since the cessation of the original FAUNMAP effort, many new radiocarbon dates from existing sites have been published, and many new dated fossil localities have been added to the database. An update to the chronologies underlying the FAUNMAP constituent database is long overdue. We therefore undertook a revision of North American vertebrate radiocarbon chronologies in Neotoma with the aim of producing a consistent set of age estimates for Pleistocene to Holocene vertebrate fossil assemblages, suitable for use in broad-scale paleoecological studies.

We focus specifically on localities that include records of small mammals, defined here as mammals in the orders Rodentia, Lagomorpha, and Eulipotyphla, and that have at least one radiocarbon or other geochronological age estimate rather than only biostratigraphic or other age estimates. Most of the available geochronological ages are radiocarbon dates, which have a maximum age of ~55,000 ^14^C BP^16^; we have therefore restricted the temporal range of our update to the Late Pleistocene to Holocene. We focus specifically on locality-level chronologies that provide age estimates for all samples recovered from a locality. While generating chronologies based on individual specimens is now standard for many single-species studies focused on, e.g., estimating the timing of extinction or population expansion, these approaches tend to focus on larger mammals with large amounts of fossil material available for dating^28–31^ and are not easily applied to individual localities. Many Quaternary localities contain abundant small mammal fossils, and generating individual dates for all taxa within an assemblage is often impractical; thus inferences of age for small mammals often rely on indirect dates and inferred chronologies for the assemblage (e.g.^27,32,33^). Small mammals are abundant, diverse, and pervasive in terrestrial ecosystems; their ubiquity and trophic importance makes them a good choice for paleoecological studies comparing large numbers of fossil assemblages. The improved chronologies resulting from this effort will be of use to future researchers studying many aspects of vertebrate assemblages across the Pleistocene to Holocene climatic transition.

## Methods

**Fig. 1:**
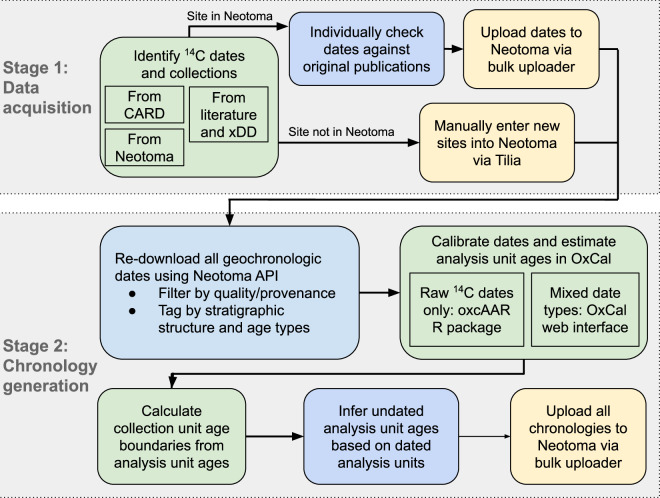
Workflow diagram summarizing the main steps in updating and augmenting radiocarbon dates to sites in the Neotoma Paleoecology Database and generating updated chronologies. Stage 1 gathers data from multiple sources (including Neotoma), cleans data, resolves transcription errors and improves the data holdings within Neotoma. Because Neotoma provides additional collection, geographic and chronological metadata we ensure that data is submitted to Neotoma to ensure additional long term curation. Stage 2 uses the existing Neotoma APIs to download the full geochronological metadata for individual records. This allows the script to be run in the future, independently of Stage 1 as new records are added or as new statistical models are developed for chronology construction. Green boxes indicate steps that were done using R scripts; steps in blue boxes were done manually; and yellow boxes indicate steps that involved the upload of data into Neotoma.

This data update consisted of two stages (Fig. 1):

1. Updates to, and augmentation of, radiocarbon dates in Neotoma
2. Estimation of new chronologies for vertebrate localities in Neotoma

### Data acquisition

We undertook a data discovery and mobilization effort to first identify existing dated vertebrate localities in Neotoma, then update and augment these sites based on 1) the contents of the Canadian Archeological Radiocarbon Database (CARD) and 2) directly searching the literature for new ages from undated small mammal sites in Neotoma.

#### Data sources

##### Neotoma

**Fig. 2:**
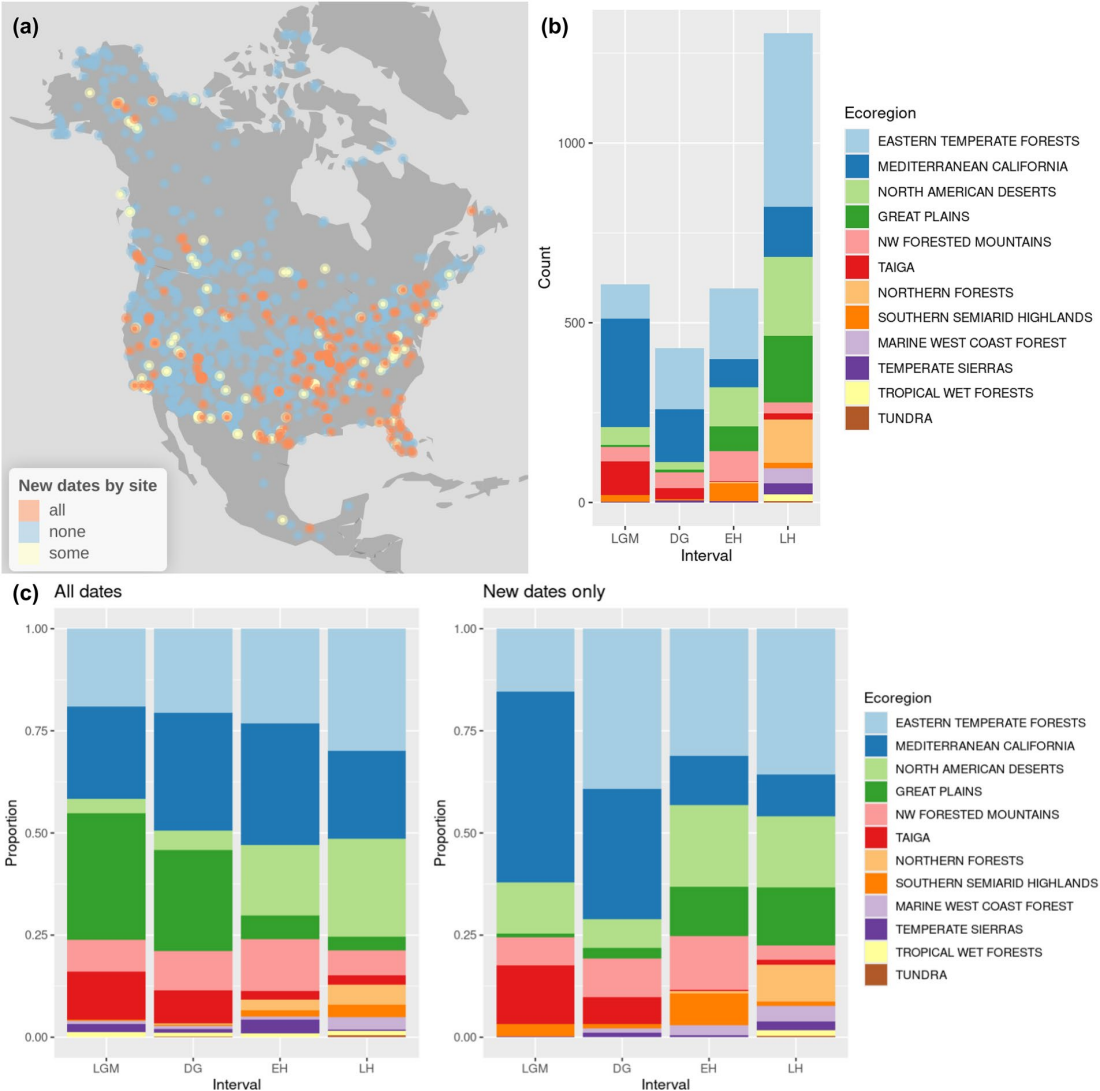
New dates added to Neotoma from literature and CARD database. (**a**) Map of all sites in this project. Blue = no new dates found; cream = some dates corrected or newly added; orange = all new dates (i.e. previously undated site). (**b**) Number of new dates added in this project for each modern ecoregion (EPA level 1 55) and time intervals. Time interval abbreviations: LGM (Last Glacial Maximum, 30,000 - 16,000 cal BP); DG (“deglacial”, 16,000 -11,700 cal BP); EH (Early Holocene, 11,700 - 6,000 cal BP); LH (Late Holocene, 6000 - 0 cal BP). (**c**) Proportional representation of EPA ecoregions and time intervals among all dates used in chronology construction in this project (left), compared to the same for new dates only (right).

The focus of our effort was on late Quaternary vertebrate localities within the Neotoma Paleoecology Database. Neotoma comprises multiple constituent databases, which typically focus on a particular proxy and/or region. Most of the data in this effort are curated within the FAUNMAP constituent database, but a subset of the sites are associated with the Alaskan Archeofaunas, PaleoVertebrates of Latin America, or other constituent databases. Relevant sites were selected from Neotoma according to the following procedure: We first downloaded a list of all sites with either vertebrate fossils or geochronological data within a polygon defining North America. From those sites, we then selected all collection units containing both vertebrate fauna and at least one geochronological date with a minimum (younger) 1-sigma age estimate (age minus error) less than 30,000 ^14^C BP years ago. The list of remaining sites was filtered to include only those collection units whose vertebrate faunas contained fossil specimens identified to taxa belonging to the mammalian orders Rodentia, Lagomorpha, and Eulipotyphla. We then assembled the full list of geochronologic dates from these collection units. The workflow described above returns 5673 dates from Neotoma (as of 24 January 2025), 5411 of which were radiocarbon dates, from 1361 FAUNMAP collection units that contained small mammal fossils (Fig. 2). Complete R code for this procedure is contained in the project Github repository (see “Code availability”).

Canadian Archeological Radiocarbon Database (CARD)/p3k14c. CARD^34^ is a database of over 120,000 radiocarbon dates from archaeological and geological sites around the world, although the focus has historically been on North American archaeology^35,36^. Lists of dates from CARD in Canada, the United States (US), and Mexico were downloaded, and the list of full references was requested from site maintainer (author A.M.). CARD is both a user-populated database and the result of regional research initiatives (e.g.^36^). Dates that were assigned to the US but not to a particular state were excluded. The two datasets of radiocarbon samples were first matched by whether the lab number was present in both CARD and Neotoma, and then by the uncalibrated (^14^C years) age and error values. If the age, error, and lab number were identical, the date was marked as correct and present in both databases. If the lab number and uncalibrated age and error values were not identical, the record was flagged for review. 408 such conflicting age or error values were checked against the original references before being added to the final table of dates to be uploaded (see “Data quality control” below.)

All remaining dates from CARD were matched against existing Neotoma sites where possible, using a variety of non-required or non-unique identifying information: machine numbers from the FAUNMAP project^26,27^, government-designated archeological site numbers (e.g. Smithsonian trinomial^37^ or Borden system number^38^), site names (using custom regular expressions to fuzzy-match names), and/or geographic proximity of coordinates (within a 50-meter radius of an existing Neotoma site). Each of these pieces of information might individually fail to identify a matching pair of sites for a variety of reasons, but in combination, they proved to be sufficient (SJG, VJPS, JLB *et al*., in prep.). The list of possible matches was then manually reviewed to identify which sites were genuinely present in both databases. We only added dates from CARD to Neotoma if they either matched an existing site in Neotoma with small mammal data, or if the date or site in CARD contained the names of taxa within Rodentia, Lagomorpha, and Eulipotyphla in the CARD fields *taxa_dated* or *associated_taxa*. In the latter case, we also added any new taxa to the Neotoma vertebrate fauna dataset separately.

Potential dates from identified shared sites were then checked against the original references. The abbreviated references recorded in the main CARD database were identified in the CARD reference list where possible, or using WorldCat or Google Scholar if necessary. 209 dates with unidentifiable or inaccessible references were dropped. Coding of raw ^14^C ages as “measured age” versus “normalized age” in CARD was inconsistent, so the date type was checked in the references and entered as described in the original publications. Based on the historical and stratigraphic context recorded in CARD or the original references, dates were assigned in Neotoma to either an existing analysis unit, a new analysis unit belonging to an existing collection unit, or a new collection unit at the site. In total, 1,187 dates were found and verified by this process.

Within CARD, publicly-available site locations of dates from the US were obfuscated to the county level in order to safeguard potentially sensitive locations. In order to avoid exposing these data through Neotoma, we dropped 1,098 dates that came from 139 sites whose locations were obfuscated in CARD to a lower precision than their location in Neotoma. We recognize that this is a compromise that means that some sites are spatially resolved more finely in Neotoma than in CARD; though we have not added new dates to these sites, the sites still exist in their original spatial resolution in Neotoma. Neotoma leadership (including author J.L.B.) is currently focused on identifying sites within Neotoma for which the current spatial resolution is not appropriate and implementing changes to the spatial precision of some sites. In the future, if site spatial precision is changed to a resolution that matches CARD, we will upload the newly identified dates and update their chronologies following the process outlined here.

Overall, we uploaded 89 dates from 16 collection units from CARD into Neotoma. The p3k14c database^39^, which incorporated CARD, was accessed using the *p3k14c* R package^40^ in order to check for additional dates. All relevant dates in p3k14c were either present in Neotoma already or were originally from CARD and therefore had been captured by the process detailed above.

##### Literature search

Additional dates were added from the literature based on a search using xDD^41^, the journal *Radiocarbon*, and Google Scholar. We focused our efforts on sites already in Neotoma for which focal taxon occurrences were recorded, but for which there were no geochronological dates. We compiled a list of potential sites, and then each of these site names was searched against xDD along with the dictionary terms “radiocarbon” and “North America”, using the “snippets” API tool. Each site name was also searched against the radiocarbon date lists published in *Radiocarbon*, which is not indexed in xDD but has open-access machine-readable PDFs available on the journal website. Remaining undated small mammal sites were individually searched in Google Scholar. All references returned by any of these search methods were checked manually for any dates not already present in Neotoma. These searches resulted in the addition of 1,985 radiocarbon dates for collection units already in Neotoma. Searches also yielded new small mammal records for 22 existing collection units, 36 new dated sites with small mammal fossils, and a number of corrections to previously recorded dates. The full process is documented in the code in the project Github repository.

#### Date validation and quality control

Neotoma supports the use of 19 different date types, including geochronologic, stratigraphic, and event-based date types. The final small mammal chronology dataset included 7695 uncalibrated radiocarbon dates, as well as 92 radiocarbon dates for which the uncalibrated date could not be found, 51 thermoluminescence, 34 uranium-series, 1 argon-argon, 4 optically stimulated luminescence, and 2 paleomagnetic dates. OxCal can incorporate date types other than uncalibrated radiocarbon into composite chronological models by coding them as calendar dates (C_Date). We added the non-radiocarbon dates into the OxCal age model as calendar dates for collection units containing both radiocarbon and non-radiocarbon dates. The resulting age models were computed for the other multi-date collections and interpreted as described in “Computing age estimates”. No new chronologies were computed for analysis units without any uncalibrated radiocarbon dates.

##### Error checking

Dates present in both CARD and Neotoma were checked against the original publication. Of these, 214 dates had incorrect or missing mean ages in Neotoma; 22 of these had additional metadata errors. All such records were corrected in Neotoma. Some dates were incorrect or incomplete in both databases when compared to the original publications; these were also corrected in Neotoma and submitted to CARD. 12 dates in Neotoma or CARD had asymmetric error values (e.g. “1200, −110/ + 70”). In all these cases, we located the original error values via literature search and confirmed that they were originally reported as uncalibrated values with asymmetric errors, and retained them in the database in this form. For 208 dates in CARD, either no original publication could be identified or the original publication was not available online or via the University of California library system, including dates for eight sites with no other identified dates. However, as all eight of these sites were from the Late Holocene, ≤6ka (for which data are available from many other sites), and the remainder of the untraceable dates were from already-dated sites, we did not pursue them further. We did not retain dates for which the original publication or the CARD notes indicated they were derived from human remains. Note that not all sites with vertebrate fauna data in Neotoma were checked, only those from collections that contained small mammal fossils and radiocarbon dates ≤30,000 ^14^C BP; errors may remain in Neotoma collections older than 30 ka and/or not containing small mammals.

Revisiting the original publications as part of quality control revealed 58 dated small mammal fossil collections from new sites not previously included in Neotoma, as well as a number of small mammal fossils belonging to existing Neotoma collection units but not previously included in the taxon lists for those sites in Neotoma. These data were individually added to the database. Finally, two sites in our list, Lubbock Lake and Natural Trap Cave, have undergone significant recent stratigraphic revisions and redating, and have been omitted from this effort due to the complex revisions needed to their data structure in Neotoma.

##### Date types and qualities

Experimental determination of radiocarbon dates is known to be affected by the choice of original material, sample treatment, and dating method^5,42,43^. The effects of these factors on ^14^C date precision and accuracy have been documented in depth. In general, for the purpose of dating small mammal fossil sites, the ideal original material would be bone from a specimen of the particular species of interest; the optimal sample pretreatment for bone samples would be collagen extraction and XAD ultrafiltration^44,45^; and the modern standard for dating method is accelerator mass spectrometer (AMS) dating^42,46,47^. Unfortunately, only a handful of the dates in Neotoma are documented as meeting these standards. Of the 7695 radiocarbon dates in our final data set, 2579 have no dating method entered, and only 809 are labeled as AMS. The material dated mentions “bone” for 1950 dates and “collagen” or “gelatin” (presumably from bone) for 829. Filtering, purification, or extraction, including XAD, is mentioned in the metadata for only 72 dates; the vast majority of dates do not have metadata on sample pretreatment. Additionally, the 284 dates missing their original lab numbers are effectively untraceable and therefore must also be regarded as less reliable. If we restrict our data set to only AMS dates with known lab numbers from XAD-extracted bone collagen, we are left with a total of 18 dates and 7 dateable collections.

As our goal is to provide a large set of chronologies suitable for regional- or continental-scale analyses of species distributions or comparisons to other data sets, we have chosen to prioritize breadth over precision by including all dates accepted by the original authors in our chronology revision, including low-quality and/or poorly-documented dates. This includes dates with missing metadata and outdated methods, as well as those derived from materials such as plant charcoal^48^ or insect chitin^49^, which may tend to be older or younger than co-occurring small mammal fossils. The potential uses of a wide-ranging, homogeneous, but lower-precision set of chronologies are discussed further in the section “Usage notes”.

### Computing new age estimates

In order to generate new chronologies, we started with a fresh date list by using the *neotoma2* R package^50^ to download all the geochronological dates from collections with small mammal fossils in North America and current age estimates <30,000 ^14^C BP, including both previous and newly-updated dates (total n = 8459). We then filtered the dataset to eliminate 183 radiocarbon dates that were single-sided (lower age limit only, e.g. “ >31,500”) or modern (age estimate ≤10 cal BP), 303 with missing or zero error values, 314 for which the *materialdated* or *notes* fields indicated that the age estimate had been rejected by the original author, and 9 for which the *materialdated* or *notes* fields indicated that the sample came from human remains (categories may overlap). This eliminated 28 collections lacking reliable or usable radiocarbon dates. We additionally dropped 32 collections that had no radiocarbon dates at all. The final data set used for age estimation consisted of 7695 radiocarbon dates and 92 other dates, allowing us to compute chronologies for 1551 collection units from 1382 sites.

**Fig. 3:**
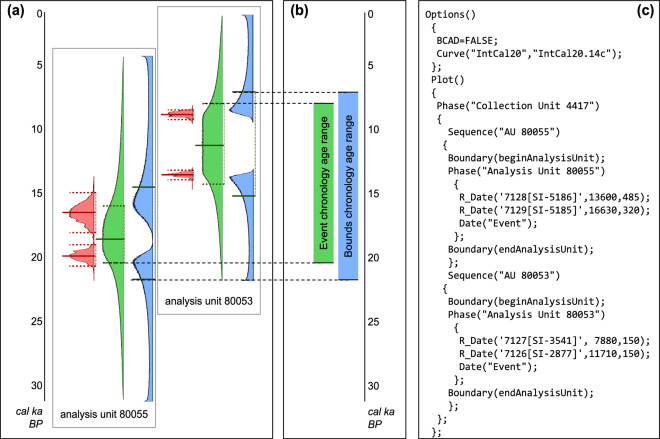
Example chronology. This collection unit belongs to the Neotoma site “Selby” from eastern Colorado (siteid: 3534) and can be viewed in Neotoma Explorer using that name (or the dataset landing pages data.neotomadb.org/4562 and data.neotomadb.org/9273). It consists of two analysis units with no recorded stratigraphic order, each containing two radiocarbon dates. (**a**) Calibrated radiocarbon dates and analysis unit age estimates, plotted on a vertical age axis. Red curves show the calibrated age distributions of the individual 14C dates; solid lines indicate medians and dotted lines indicate 5% and 95% quantiles. Green curves show the estimated sample age distribution for each of the two analysis units; solid lines indicate medians and dotted lines indicate 32% and 68% quantiles. Blue curves show the estimated upper and lower age boundaries for each analysis unit; solid lines indicate medians. (**b**) Collection-unit-level age boundaries for each of the two chronology types based on the age distributions in (**a**), plotted on the same vertical age axis and using the same colors. As indicated by the dotted lines connecting (**b**) to (**a**), the *event* chronology (in green) is bounded by the maximum and minimum 32%/68% quantiles of the event age distributions; the *bounds* chronology (in blue) is bounded by the maximum and minimum 50% quantiles of the boundary age distributions. (**c**) Automatically-generated OxCal code used to calculate the example chronology in Fig. 2.

We then used OxCal (v. 4.4)^51^ to compute an age model for each collection unit in the data set (Fig. 3a,b), using a script that automatically computed OxCal scripts (Fig. 3c), as follows:

1. All geochronological dates in each analysis unit were grouped into a single phase using the Phase() function, to capture the time horizon over which depositional processes formed the analysis unit. Uncalibrated ^14^C dates, which make up the majority of the data set, were calibrated using R_Date(). Pre-calibrated or non-radiocarbon dates were incorporated using C_Date(), as discussed under “Date types” below. The phase for a single analysis unit could contain one or multiple dates.
2. Two different age estimates were calculated for each analysis unit; one used a Date() call inside the phase and the other used two Boundary() calls outside the phase. All age distributions were estimated based on uniform prior distributions. The difference between these two types of age distribution model, and the rationale for calculating both, is discussed under “Dated analysis units” below.
3. Each collection unit was modeled as a single Sequence() unit in order to constrain the Bayesian age estimates, with one phase for each analysis unit. If the analysis unit names or depths indicated a clear stratigraphic order, they were ordered from oldest to youngest, and the whole collection was modeled with Sequence(); otherwise, it was modeled as a phase with no ordering. (Note that the overall collection unit age boundaries were *not* separately modeled; see “Overall chronology age estimates” for details).

#### Estimating sample ages for analysis units

Chronologies are reported in Neotoma as the individual control points on which the sample ages are based (Fig. 3a, red curves), the estimated age range of each analysis unit (‘sample age’) in the collection (Fig. 3a, blue and green curves), and the maximum and minimum age of the overall collection unit (Fig. 3b). Our procedure for estimation of sample ages consisted of first estimating the ages of analysis units with geochronologic dates, then combining those estimates with stratigraphic context to infer the ages of undated analysis units in the same collection. We developed two different chronologies for each collection unit, corresponding to two different methods for inferring the sample ages, which we named the ‘event’ and ‘bounds’ chronologies. The difference in methods between the two is detailed below.

##### Dated analysis units

For analysis units with at least one geochronologic date, the sample ages were directly calculated as quantiles of the age probability distributions generated by OxCal (Table 1).

**Table 1:** Quantiles of the Bayesian age distribution estimates used to define sample age values for each of the two new sets of chronologies.

chronology name	NeotomaDB field		
	*sampleages.agelimitolder*	*sampleages.age*	*sampleages.agelimityounger*
**bounds**	0.5 quantile of upper bound age distribution	[none]	0.5 quantile of lower bound age distribution
**event**	0.3173 quantile of event age distribution	0.5 quantile of event age distribution	0.6827 quantile of event age distribution

The sample age estimate for each directly dated analysis unit in the ‘event’ chronology was based on the OxCal estimate of the age distribution for that analysis unit; it can be thought of as the expected value of a new date drawn from the same pool as all the dates currently in the analysis unit^15,51^. The age estimates for the ‘bounds’ chronology, by contrast, was based on the OxCal estimates of the age distribution for the minimum and maximum ages of the dated analysis unit, and therefore can be thought of as the estimated starting and ending dates for the deposition of the analysis unit. From a researcher’s perspective, there are advantages to each. In general, the bounds chronology provides a more conservative estimate of unit age for time-averaged deposits, and thus produces a more consistent result between collections with a single date and collections with more complex structures. The event chronology provides a statistically meaningful central estimate of collection age, but we found that it is more sensitive to the number of dates and stratigraphic units in the collection.

In choosing which quantiles to report from the different estimated age distributions, we have attempted to balance a variety of priorities: limiting sensitivity to the number of dates, and providing a usable but not misleading level of precision. However, we recognize that some arbitrariness is unavoidable in the choice of how to report discrete values from continuous distributions. For the bounds chronologies, highly outward-skewed distributions are characteristic of the boundary age estimates (see Fig. 3); the medians (50% quantiles) of the boundary age estimates provide a conservative estimate of the depositional window for the collection, but largely avoid this statistical artifact. The event age distributions, while typically not highly skewed, are often platykurtic, falling somewhere between normal and uniform; our choice to report the 32% and 68% quantiles is intended to dampen the sensitivity of this measure to sparse data and outliers.

##### Undated analysis units

For collection units containing some dated and some undated analysis units, the results were individually manually examined, and the age estimates for the dated samples (analysis units) were used to infer ages for the undated samples. Many analysis units correspond to stratigraphic units; stratigraphic relationships can therefore be used to constrain the possible ages of some of the undated samples.

**Fig. 4:**
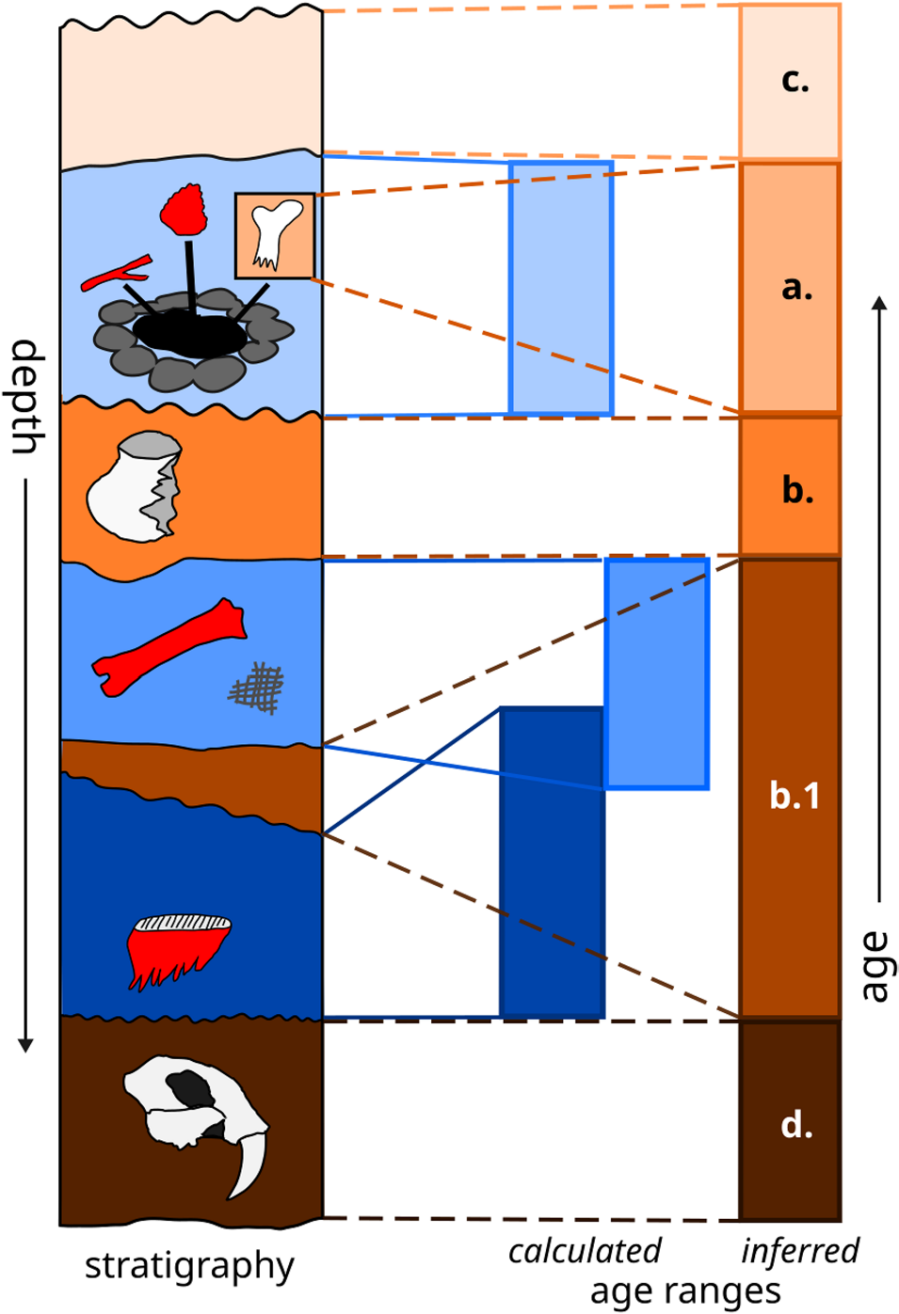
Summary of rules used to infer the ages of undated analysis units in a collection from ages of dated units. The column on the left marked “stratigraphy” shows the stratigraphic field relationships of all analysis units in the collection unit, as well as the presence of 14 C-dated specimens (red filled outlines). Blue-shaded analysis units indicate analysis units containing 14 C-dated specimens; orange shading indicates analysis units without direct dates, whose ages must therefore be inferred from stratigraphic relationships. The same shades are used for the corresponding analysis unit ages on the right side, marked “age ranges”. Calculated analysis unit age ranges (blue shaded) are based on 14 C-dated specimens, as described in the section “Dated analysis units”, and are connected to the corresponding strata with solid lines. Inferred analysis unit age ranges (orange shaded) are derived from calculated ages based on stratigraphic relationships, and are connected to the corresponding strata with dotted lines; lowercase letters in each analysis unit indicate which rule was used to infer the age of that unit: (**a**.) subset of dated layer; (**b**.) bounded above and below by dated layers; (**b**.1) bounded by overlapping dated layers; (**c**.) above all dated layers; (**d**.) below all dated layers. These heuristics are described in more detail in the section “Undated analysis units”.

The inferred sample age boundaries for the undated analysis units in each collection unit were assigned according to the following heuristics, graphically summarized in Fig. 4:

- If an undated analysis unit was a **subset** of a dated analysis unit (e.g., a single undated specimen from a dated stratum), it was assigned the age range of that dated unit.
- An undated analysis unit **bounded above and below** by dated analysis units was assigned a maximum (oldest) age from the top of the next dated unit below it and the minimum (youngest) age from the bottom of the next dated unit above it.

1. If the age ranges of the two dated units overlapped, it was assumed that time-averaging had occurred, so the undated unit was instead assigned the full range of the dated analysis units above and below it.

c. An undated analysis unit stratigraphically **above** all dated analysis units was assigned a maximum (older) age of the top of the next dated unit below it and a minimum age of 0, unless other biostratigraphic or archeological information allowed its minimum age to be further constrained.
d. An undated analysis unit stratigraphically **below** all dated analysis units was assigned a minimum (younger) age of the bottom of the next dated unit above it and a maximum (older) age based on any biostratigraphic or archeological constraints recorded in existing chronologies or, if no information about this is available in Neotoma, in the original publication.
e. If an undated analysis unit had **no apparent stratigraphic relationship** to the other units in the collection, it was assigned the full age range of all dated units in the collection, i.e. the same maximum and minimum age as the whole collection unit.
f. No central age values were estimated for undated analysis units.

These rules were used to assign inferred age ranges for a total of 926 undated analysis units belonging to 197 of the collection units.

#### Overall chronology age bounds

The age range for any one collection unit is defined as the oldest and youngest ages inferred for all analysis units within the chronology, including analysis units that extend to the surface (set to the standard ^14^C “year zero”, 1950 CE) or analysis units below ^14^C-dated units with maximum age estimates based on stratigraphic, biostratigraphic, or archeological data (i.e. orange-shaded analysis units in Fig. 4). Both dated and undated sample ages were used in estimating the collection unit maximum and minimum ages in the Neotoma table *chronologies*.

The decision to include inferred age ranges was based on the expectation that the primary use of collection unit age ranges is for API-based data filtering. When a user of either the *neotoma2* R package or the Neotoma Explorer web interface requests sites falling within a certain age range, the API will filter the data using the collection unit age ranges in the default chronologies and return all sites with collection units that overlap the desired age range. Since users may be looking for information on fossils that pre- or post-date the range of directly dated units (in Fig. 4, the highest or lowest orange-shaded units), limiting the search to only directly dated units might exclude relevant results. We have therefore reported the maximum and minimum ages for the collection units in the *chronologies* table under the most generous interpretation possible.

## Data Records

The primary repository for the data generated in this project, consisting of the table of geochronologic dates compiled in stage 1 and the chronologies generated in stage 2, is the Neotoma Paleoecology Database, where they have been integrated into the relational structure of the database. They can be accessed from the Neotoma API as an aggregated dataset^52^. The new dates and new chronologies, exactly as uploaded at publication, are also archived in the Zenodo release of the Github repository^53,54^.

We have provided an example of how the new chronologies for a single site are represented in the Neotoma database schema (open.neotomadb.org/dbschema) in Table 2. Raw geochronologic dates (in this case uncalibrated radiocarbon ages and errors, lab identification numbers, and associated δ13C values) are stored in the table *geochronology*, and the related sample metadata (material, preparation methods, associated specimens) are linked to it via the table *samples*. The geochronologic data sets can be viewed using the Neotoma Explorer web interface or downloaded with the get_table() function in the neotoma2 R package^50^. Within Neotoma, the geochronologic dates added to the database from this project are publicly available, but have not been marked in any way that allows them to be distinguished from the other data in the table.

**Table 2:** Data uploaded to Neotoma tables for the two chronologies calculated for example collection unit 4417 in Fig. 3: (**a**) *chronologies* (**b**) *sampleages* (**c**) *chroncontrols*.

(a) Table of overall age ranges for new chronologies					
collectionunitid	agemodel	ageboundolder	ageboundyounger	dateprepared	chronologyid
4417	Bayesian unit bounds	22472	6767	2024-08-27	46965
4417	Bayesian event distribution	19797	9582	2024-08-27	46966

(b) Table linking sample (analysis unit) ages to chronologies						
chronologyid	analysisunitid	agemodel	age	ageolder	ageyounger	sampleid
46965	80053	bounds	*NA*	15604	6804	103001
46965	80053	bounds	*NA*	15604	6804	114969
46965	80053	bounds	*NA*	15604	6804	114970
46965	80054	bounds	*NA*	16857	12867	103002
46965	80055	bounds	*NA*	22424	14164	103003
46965	80055	bounds	*NA*	22424	14164	114971
46965	80055	bounds	*NA*	22424	14164	114972
46966	80053	event	11214	12854	9584	103001
46966	80053	event	11214	12854	9584	114969
46966	80053	event	11214	12854	9584	114970
46966	80054	event	*NA*	22472	6767	103002
46966	80055	event	18374	19794	16884	103003
46966	80055	event	18374	19794	16884	114971
46966	80055	event	18374	19794	16884	114972

(c) Table linking individual dates to chronologies and analysis units; note that these are the calibrated ages estimated for each ^14^C date, not the raw ^14^C ages in the Neotoma table *geochronology*							
chronid	analysisunitid	geochronid	labnumber	age	agelimitolder	agelimityounger	chroncontrolid
46965	80053	7126	SI-2877	13574	13874	13334	137777
46965	80053	7127	SI-3541	8764	9104	8454	137778
46965	80055	7129	SI-5185	20034	20724	19394	137779
46965	80055	7128	SI-5186	16654	17924	15454	137780
46966	80053	7126	SI-2877	13574	13874	13334	145568
46966	80053	7127	SI-3541	8764	9104	8454	145569
46966	80055	7129	SI-5185	20034	20724	19394	145570
46966	80055	7128	SI-5186	16654	17924	15454	145571
46966	80054	*NA*	*NA*	*NA*	22472	6767	150677
46965	80054	*NA*	*NA*	*NA*	16857	12867	150678

Permanent ID values (replacing the temporary ID values given in italics) are generated when the data are uploaded to the database. The tables here do not represent all the data fields uploaded for each collection; the fields that are identical for all chronologies have been omitted. These include the chronology name (“Syverson-Blois 2025”), the age type (Carbon-14), default status (True), and contact information. The fields ending in “id” are unique keys generated at the time of upload.

The *chronologies* table stores the chronology name, model, and the estimated maximum and minimum ages for the collection unit. It links to the constituent analysis unit ages (maximum, minimum, and central) in the *sampleages* table, and to the chronology control points (calibrated radiocarbon ages and all other age estimates or dates used in chronology construction) in the *chroncontrols* table. Each radiocarbon-based chronology control point is referred back to its corresponding raw geochronologic dates in *geochronology* via the linking table *geochroncontrols*. For further details of chronology definition in Neotoma, see Williams *et al*.^24^ and the Neotoma manual (open.neotomadb.org/manual). For the relational structure of all tables linked to chronologies, see open.neotomadb.org/dbschema/ndb/tables/chronologies.html, and for tables linked to geochronological dates, see open.neotomadb.org/dbschema/ndb/tables/geochronology.html.

The chronologies from this project have the chronology name “Syverson-Blois: bounds” or “Syverson-Blois: event” and the age model names “Bayesian unit bounds” or “Bayesian event distribution” in the *chronologies* table. Each individual chronological control (i.e. entry in the *chroncontrols* table) for each chronology is linked via the *sampleages* table to a particular date in the *geochronology* table. The set of chronologies for any site in Neotoma, including the top-level collection unit age boundaries and all constituent analysis unit ages and control points, can be viewed using the function “chronologies” in the *neotoma2* R package^50^. The “Syverson-Blois: bounds” chronologies have been set as the default chronologies for all sites included in this update, which means that they are also returned by default in the Neotoma Explorer “samples” tab and dataset download and automatically used for age-based queries from the API.

The Zenodo-archived Github repository contains the updated dates^53^ and chronologies^54^ unlinked from the rest of the Neotoma relational database. It also contains seven RMarkdown notebooks containing the entire workflow, as well as various.csv files containing data and R scripts containing functions that are called from the notebooks. Scripts 1–5 guide the user through the process of downloading geochronological dates from Neotoma and generating chronologies for all Late Pleistocene through Holocene small mammal collections, as was done in this project. Script 6 is a concise version of the previous five scripts that is designed to retrieve the current chronological and taxonomic information for all collections in a single site or a small set of sites specified by their Neotoma site ID, and is likely to be the most useful to any readers interested in recomputing the chronology for particular collections, for instance if they have added more radiocarbon dates to the database for their study site. Script 7 uses the outputs of the other scripts to generate the figures in this paper. All code was written by VJPS on Debian Linux 12 and tested by JLB on Mac OS 15, using R v4.2.0 or later with libraries neotoma2 v1.0.5^50^ and oxcAAR v1.1^51^.

## Technical Validation

### New and updated radiocarbon dates

Following the cleaning procedures, we made the following improvements^52,53^ to the available set of radiocarbon dates for small mammal sites in Neotoma (Fig. 2):

- Added 2074 new dates to 264 collections at 254 existing sites
- Corrected or updated 452 dates already in Neotoma
- Added 42 new sites with dates
- Updated 22 existing collection units with new small mammal fossil occurrences

To examine the effects of our update on temporal coverage, we binned the dates into four intervals, designated LH (Late Holocene, 6000 - 0 cal BP), EH (Early Holocene, 6,000–11,700 cal BP), DG (“deglacial”, 11,700–16,000 cal BP), and LGM (Last Glacial Maximum, 16,000–30,000 cal BP). The majority of dates added were from the Late Holocene, as is true of the overall data set we used for computing the new chronologies. However, we have significantly added to the number of available Pleistocene (2.588 Ma - 11,700 cal BP) dates with this update, increasing the number of LGM dates by nearly 70% and more than doubling the number of DG dates. The distribution of dates in the dataset is significantly influenced by geographic bias in collection and data upload, most obviously evident in the underrepresentation of fossil sites from Mexico. Taphonomic factors also influence the availability of dates; for example, northern areas covered by continental ice sheets at the Last Glacial Maximum lack Pleistocene dates. The representation of Level 1 EPA ecoregions^55^ is also temporally uneven; for example, dates from Great Plains and Northern forest sites are overrepresented in the Holocene, while most dates from taiga environments are of Pleistocene age. This largely reflects known taphonomic and sampling biases (Bellvé *et al*., in review.). A cross-tabulation of ecoregions and time periods is given in Fig. 2c.

Our sampling effort compiles legacy data from original literature dating back to the earliest published radiocarbon lab date lists, which confines its scope to those dates that were at some point published in accessible literature with sufficient metadata for identification. Updates to some sites or dates based on later work may have occurred in the museum context but never been published in any public-facing literature; these would not be captured by our process. Additionally, some dates may have been missed by our process due to inconsistencies in the metadata used to identify sites and dates (e.g., sites with multiple names, differences between paleontological and archeological stratigraphic practices, failure to report lab numbers) and in the date values themselves (e.g., failure to report reservoir correction or calibration status). These inconsistencies should be taken as motivation for better standardization of date reporting and application of permanent identifiers to radiocarbon dates in order to improve interoperability between museum repositories, authors and scientific publishers, and online aggregators like Neotoma^56^ (SJG, VJPS, JLB *et al*., in prep.).

### Updated chronologies

**Fig. 5:**
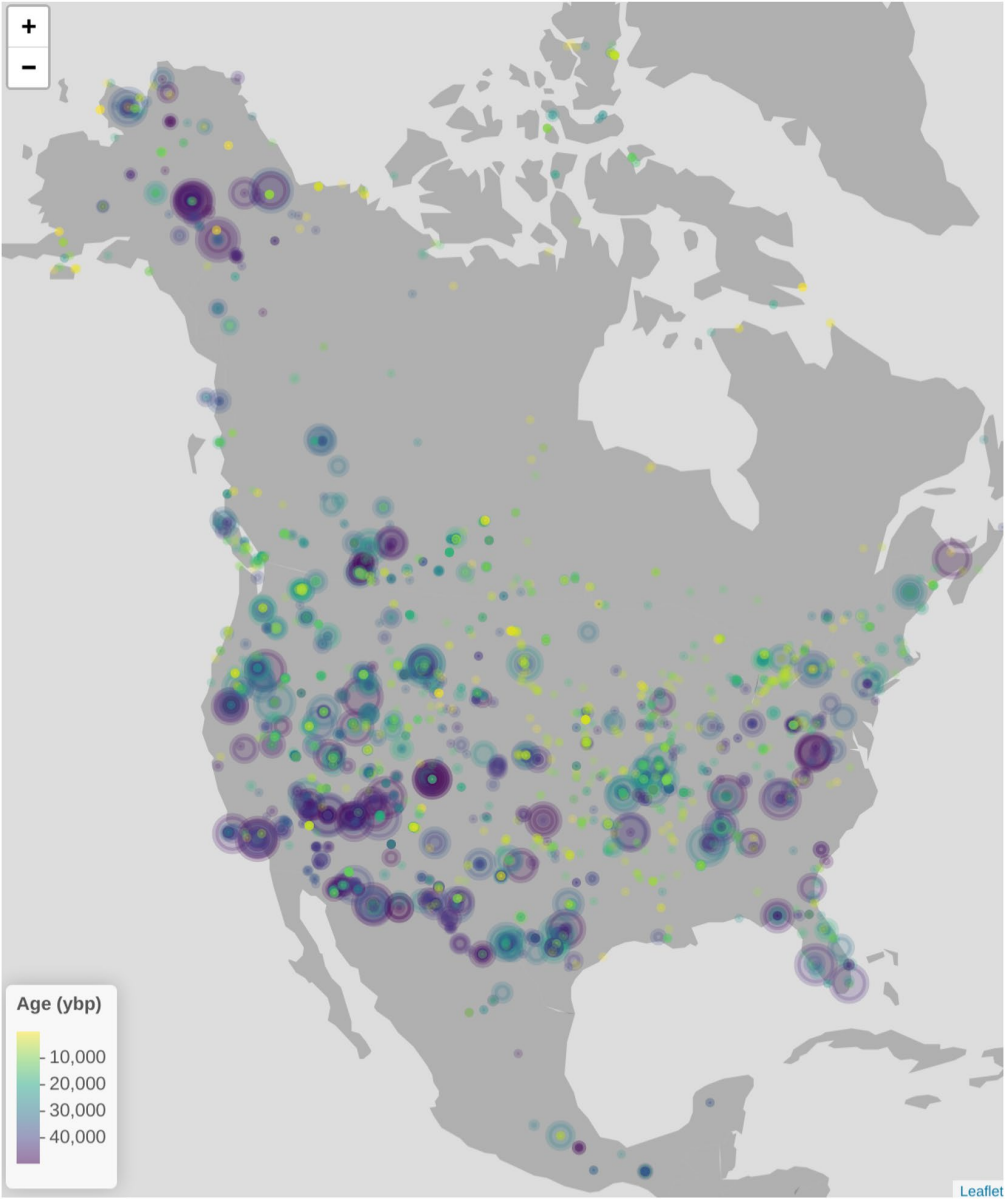
All analysis unit age estimates from the radiocarbon chronologies calculated in this project (event chronology only). Each circle corresponds to one analysis unit; multiple concentric circles indicate several analysis units from the same site. Color indicates median age of the analysis unit, as shown in legend; roughly, yellow dots are Late Holocene, green are Early Holocene to Deglacial, and blue/purple are Pleistocene. Circle size indicates age precision, with larger circles indicating analysis units with wider estimated age boundaries; the largest circles shown correspond to ~1 kyr age ranges.

We computed and uploaded new calibrated radiocarbon chronologies^52,54^ for a total of 3648 analysis units from 1,553 collection units containing small vertebrate fossils. 2,731 analysis units were directly dated from samples, while age estimates for a further 928 analysis units were inferred based on their stratigraphic relationships. 67 of the collection units with new chronologies from this project had no existing chronology in Neotoma; of the remaining 1,486 collection units, 1,158 of them previously had default chronologies based on uncalibrated radiocarbon dates, and 333 were based on calibrated radiocarbon age or other calendar age methods. While more work remains, this update significantly improves standardization and completeness of the vertebrate faunal sites in Neotoma. The geographic locations and age ranges of the new analysis unit age ranges are summarized in Fig. 5.

**Fig. 6:**
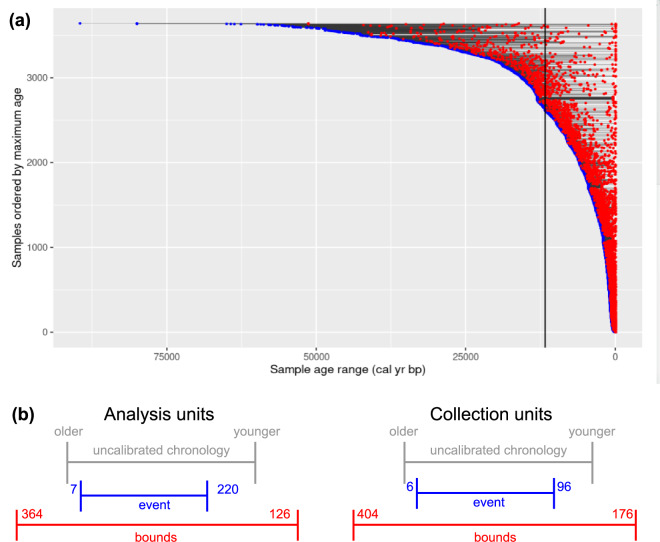
Graphical comparisons of age ranges between chronologies. (**a**) Plot of distribution of analysis units age ranges (bounds chronology only). For each analysis unit, its maximum calculated age is indicated with a blue dot, minimum age with a red dot, and the two are connected with a horizontal black line. Vertical black line indicates the Pleistocene-Holocene boundary (11,700 cal BP). Eight units with maximum sample ages >150,000 cal BP have been omitted from this plot for visual clarity. Note that while age ranges are on average much larger for older analysis units, many Pleistocene and early Holocene units are dated to relatively high precision. (**b**) Graphical depiction of data in Table 2, comparing the mean differences in older and younger age boundary estimates between the previous (uncalibrated) default chronologies and the two new chronologies described in this document, for both dated analysis units (left) and collection units (right). Compared to the uncalibrated chronologies, the *bounds* chronologies are generally less precise and the *event* chronologies are more precise; also, both new chronologies skew older than the previous chronologies due to the calibration.

For those collections and analysis units that previously relied on uncalibrated radiocarbon chronologies, we can directly assess the effects of date calibration and Bayesian modeling of age distributions (Table 3; Fig. 6). Calibrating the radiocarbon ages, as expected from the fact that the best-fit slope of the intcal20 curve is less than −1, increases the central age estimate for each dated analysis unit by a median of 7 years for those analysis units with existing median age estimates. Compared to the previous uncalibrated chronologies, our re-estimation of the analysis unit age ranges decreased the ranges of the analysis units by a median of 198 years using the “event” method and increased them by a median of 490 years using the “bounds” method (Fig. 6a). Results are similar for the overall chronology (collection unit) age bounds: the new “event” chronologies start on average 6 years later and end 96 years earlier than the old ones, while the new “bounds” chronologies extend a median of 404 years before and 176 years after the old uncalibrated chronology bounds (Fig. 6b). While the “bounds” chronology does downgrade the apparent precision of sample age estimates, this more conservative estimate more closely reflects realistic limits to the accuracy with which sample ages can be inferred from a small number of time-averaged dates.

**Table 3:** Differences between new “event” and “bounds” chronologies (in cal BP) and previous default Neotoma chronologies (in ^14^C BP).

Chronology name	Median difference from previous (uncalibrated ^14^C) chronologies				
	Analysis units (samples)			Collection units	
	Age	Older bound	Younger bound	Older bound	Younger bound
event	+7	−2	+220	−6	+96
bounds	NA	+364	−126	+404	−176

Values indicate the change in age: positive numbers indicate that the new chronology age estimate is older than the previous one, and vice versa.

## Usage Notes

Our chronologies have been set as the default in Neotoma because the calibrated age basis allows them to be directly compared to other datasets in the database, such as pollen cores or varve sequences, that are dated on the basis of calibrated radiocarbon or calendar age. These chronologies are not suitable for all research projects. Many studies require detailed species- or site-level chronologies based on individually dated specimens and high-quality dates with detailed stratigraphies, such as inferring extinction timing^1^. Our chronologies are intended to inform regional to continental level studies, which benefit from a uniform set of chronological inferences with reasonable error estimates.

As discussed in the subsection “Dated analysis units” above, this project produced two different chronologies for each collection unit evaluated. The “Syverson-Blois: bounds” chronology gives broader (more conservative) age ranges and does not provide a central age estimate. This chronology is recommended for most uses, and provides the best representation available of the uncertainty associated with the ages of samples within each analysis unit. The “Syverson-Blois: event” chronology provides narrower (less conservative) age ranges, and additionally gives a central age estimate for each radiocarbon-dated sample in the collection. This chronology may be suitable for applications where a single age for each analysis unit is needed, with the caveat that this usage masks the temporal uncertainty of the samples, which can be substantial in some cases. We have therefore set the “bounds” chronology as the default chronology in Neotoma, but uploaded both chronologies to Neotoma so that individual researchers can choose the chronology best suited for their research.

## Data Availability

The aggregated dataset in Neotoma, including both dates and chronologies along with all other data for the affected sites, is available for download from the Neotoma API at the endpoint api.neotomadb.org/v2.0/data/aggregatedatasets/13^52^. The newly added dates and chronologies are separately available as dates pub copy.xlsx^53^ and chronologies pub copy.xlsx^54^ in the Zenodo release of the Github repository: 10.5281/zenodo.17064489.

## Code availability

The R and OxCal code used in this project is available in the Zenodo release of the Github repository: 10.5281/zenodo.17064489.
